# Estimation of actual evapotranspiration in barley crop through a generalized linear model

**DOI:** 10.1016/j.mex.2022.101665

**Published:** 2022-03-12

**Authors:** Adán Faramiñan, Paula Olivera Rodriguez, Facundo Carmona, Mauro Holzman, Raúl Rivas, Christian Mancino

**Affiliations:** a Consejo Nacional de Investigaciones Científicas y Técnicas, Instituto de Hidrología de Llanuras “Dr. Eduardo J. Usunoff” (IHLLA), Rep. Italia 780, B7300, Azul, Argentina; b Comisión de Investigaciones Científicas de la provincia de Buenos Aires, Instituto de Hidrología de Llanuras “Dr. Eduardo J. Usunoff” (IHLLA), Tandil B7000, Argentina

**Keywords:** Soil moisture, surface energy balance, Generalized Linear Model

## Abstract

Evapotranspiration is a key variable of the water cycle. Its calculation requires several ground data that frequently are not available. This study contains a detailed method and measurements of meteorological and energy balance variables that can be used to estimate the daily actual evapotranspiration (ETa). A linear generalized model is obtained to calculate the ETa from common variables measured in meteorological stations. The method showed a good performance over a barley crop of easthern Argentine Pampas and can be applied and tested in other great plains.

Measurements of soil-plant-atmosphere are included

The routines to reproduce the method are included

The generalized method allows the calculation of daily ETa over crops and was tested over barley crops

## Graphical abstract

**Figure:**
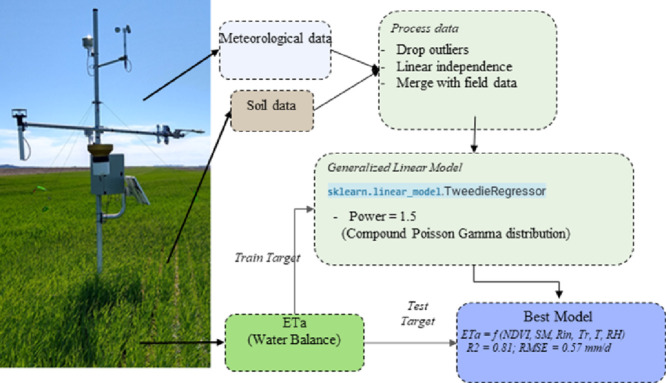

Specifications table

**Table:** 

Subject Area;	Earth and Planetary Sciences
More specific subject area;	Soil-plant water system
Method name;	Calculation of actual evapotranspiration
Name and reference of original method;	*N.A.*
Resource availability;	Dataset and routines of the model: https://data.mendeley.com/datasets/77r6w44xbp/draft?a=5d9be36a-ae9a-4c9f-b526-121d1d37b6eb

## Introduction

Evapotranspiration represents about 80% of the water transferred from soil-plant into the atmosphere over great plains [1]. The calculation of actual evapotranspiration (ETa) usually requires measurements of several variables of the atmosphere (e.g. air temperature and humidity, solar radiation) and surface (e.g. temperature, soil moisture), with nonlinear and complex interactions. The application of artificial intelligence has contributed to the estimation of the evapotranspiration, considering the fluctuations of the local climate [2]. However, given that these techniques usually limit the understanding of the relationships between the involved variables, linear generalized models (GLM) could be a possible solution. In GLM, the dependent variable is linearly related to the factors and co-variables through a link function. This study describes the method and data used to build a GLM to monitor ETa over rain-fed barley crops.

The study was carried out during the development of a barley crop (from August to December 2019 and 2020) in eastern Argentine Pampas (La Alcira station: 37,49°S, 58,90° W, 186 m.a.s.l.). The climate is temperate and subhumid. The soil type is Typic Argiudoll. Barley was sown by direct seeding with 0.17m between furrows. Ground measurements were collected from an energy balance station connected to CR1000 and CR300 data loggers (Campbell Scientific Inc.), monitoring the variables detailed in Table 1.

**Tabla 1:** Number, sensor name, variable and brand of sensors installed in the energy balance station.

number of sensor	Sensor	Variable	Brand
1	CNR4	Terms of net radiation	Campbell Scientific Inc
2	CS215	Air temperature (T) and relative humidity (RH)	Campbell Scientific Inc
1	Wind Sonic 2D	Wind speed and direction	Gill Instruments
1	014A	Wind speed	Campbell Scientific Inc
1	HFP01	Soil heat flux	Hukseflux
2	SI-111	Land surface temperature (Tr)	Apogee
3	CS655	Soil moisture and temperature (SM) at 10, 30 and 60 cm depth	Campbell Scientific Inc
1	SoilVUE10	Soil moisture and temperature at 5, 10, 20, 30, 40 and 50 cm depth	Campbell Scientific Inc
2	NR / NI	Surface reflectance in Red and NIR to calculate the NDVI	Decagon
1	TE252MM	Rainfall	Global Water

A GLM was obtained considering the most important variables for ETa estimation. The mathematical expression of the GLM is:

(1) $$Y_{i}=link\left(\beta_{0}+\sum_{j=1}^{n}\beta_{j}x_{ij}\right)$$

where *Y_i_* is the *i^th^* observation of the dependent variable (ETa), *x_ij_* is *i^th^* observation of the *j^th^* independent variable (*j = 1, 2, ..., n*), *β_j_* represents parameters to be estimated, *β_0_* is the intercept, and *link* is the link function.

On the other hand, ETa was calculated using the water balance method (WB) considering soil moisture and crop physiological characteristics. The daily WB used to calculate soil water storage considers the following terms:

(2) $$S_{f\;}-S_{i}=Ex_{f}--Ex_{i}+P--RO--DP--ET_{a}$$

where *S_f_* and *S_i_* are the final and initial soil water storage in the root zone, *Ex_f_* and *Ex_i_* are the last and initial water excess accumulated in the soil, *P* is the rainfall, *RO* is the surface runoff*,* and *DP* is the deep percolation. Subsurface horizontal water movements have been dismissed due to their values are lower than the vertical ones in plain environments. The units of Eq. 2 are in mm d^−1^. ETa was calculated as the residual term of Eq. 2 and was later used as the dependent variable of our generalized linear model.

The difference *S_f_ - S_i_* was estimated using the SOILVue10 sensor, which measures the volumetric soil moisture (SM). Water storage is directly proportional to SM [m^3^ m^−3^] times the depth covered by the sensor. We considered daily averages of *S* at a depth of 0.05-0.3 m. In the analysis period, no significant water excesses or losses due to deep percolation were recorded to calculate ETa, which can be corroborated by the water table levels in areas surrounding the experimental plot (see sheet “Water_table,” Data_GLM.xlsx, https://data.mendeley.com/datasets/77r6w44xbp/draft?a=5d9be36a-ae9a-4c9f- b526-121d1d37b6eb). On the other hand, no surface runoff was recorded during the visual control of the plot.

**Fig. 1:**
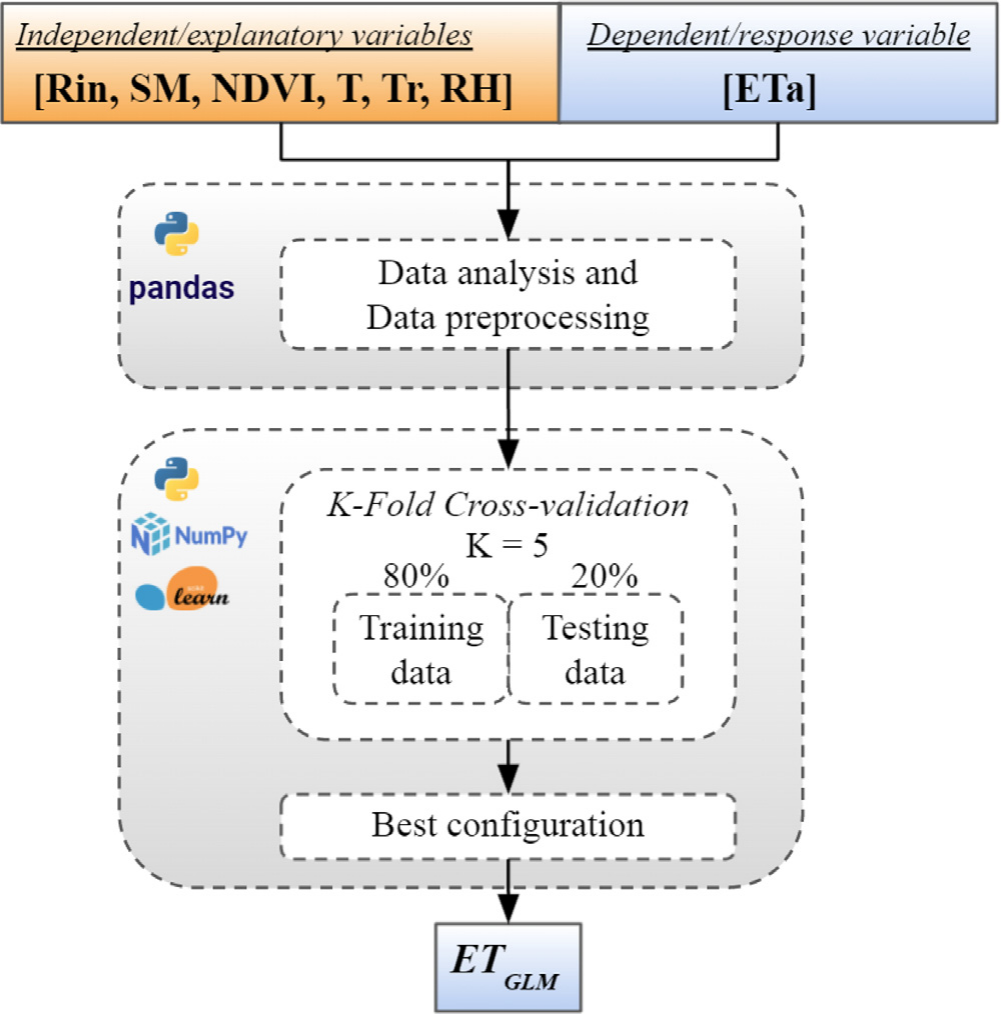
Flowchart of ET_GLM_ model.

A supervised method of learning was chosen to train the model. The flowchart of ET_GLM_ model is shown in Fig. 1. It shows the phases of the framework: i) Data collection, ii) Data analysis and data preprocessing, and iii) grid search of best hyperparameters. In step i) the response variable (ETa) and the explanatory variables were collected, where SM is the volumetric soil moisture [m^3^/m^3^], NDVI is the normalized difference vegetation index, Tr is the crop surface temperature measured at 45° [°C], Rin is the incoming (305 to 2800 nm) solar radiation at surface [MJ/m^2^/d], T [°C] and RH are the air temperature and relative humidity (%), respectively. These variables are in a daily temporal resolution.

In stage ii) the final dataset was created. Previously to build the model, an exploratory analysis of data was carried out. The variables with linear dependence (-0.7>*r of Spearman>*0.7) were discarded and the most explanatory ones (p<0.01) were selected. Statistical assumptions about the heterogeneity of variance structure and the distribution of statistical residuals were tested [3]. Finally, in step iii) a set of optimal hyperparameters for a learning algorithm was chosen in order to avoid the overfitting. For this, the K-fold cross-validation (K-CV) method was selected. K-CV is a resampling procedure used to evaluate machine learning models on a limited data sample. This technique aims to partition the training and test data about K times. K-CV is simple to understand and because it generally results in a less optimistic estimate of the model skill than other methods, such as a simple train-test split. The Python language and the Pandas [4], NumPy [5] and Scikit-Learn [6] libraries were used to develop the procedure. The routines are included in the file named MethosdX_Notebook: https://data.mendeley.com/datasets/77r6w44xbp/draft?a=5d9be36a-ae9a-4c9f-b526-121d1d37b6eb

Thus, with a total of 189 measurements, the model (mod) was obtained:

(3) $$\begin{array}{ccc} \text{mod} & = & (-4.004)+(8.4359\times \text{SM})+(2.2935\times \text{NDVI})+(0.6172\times T)\\ & & +\;(0.0926\times \text{Rin})+(-0.5486\times \text{Tr})+(-0.0227\times \text{RH}) \end{array}$$

(4) $$ETa=\;e^{\left(\;\mathrm{mod}\;\right)}$$

Rin, NDVI, T, RH and Tr were measured at 2 m height while SM was measured at 20 cm depth. It should be noted that the algorithm randomly uses the data to adjust and validate the model. The used data are included in the link https://data.mendeley.com/datasets/77r6w44xbp/draft?a=5d9be36a-ae9a-4c9f-b526-121d1d37b6ebXX

**Fig. 2:**
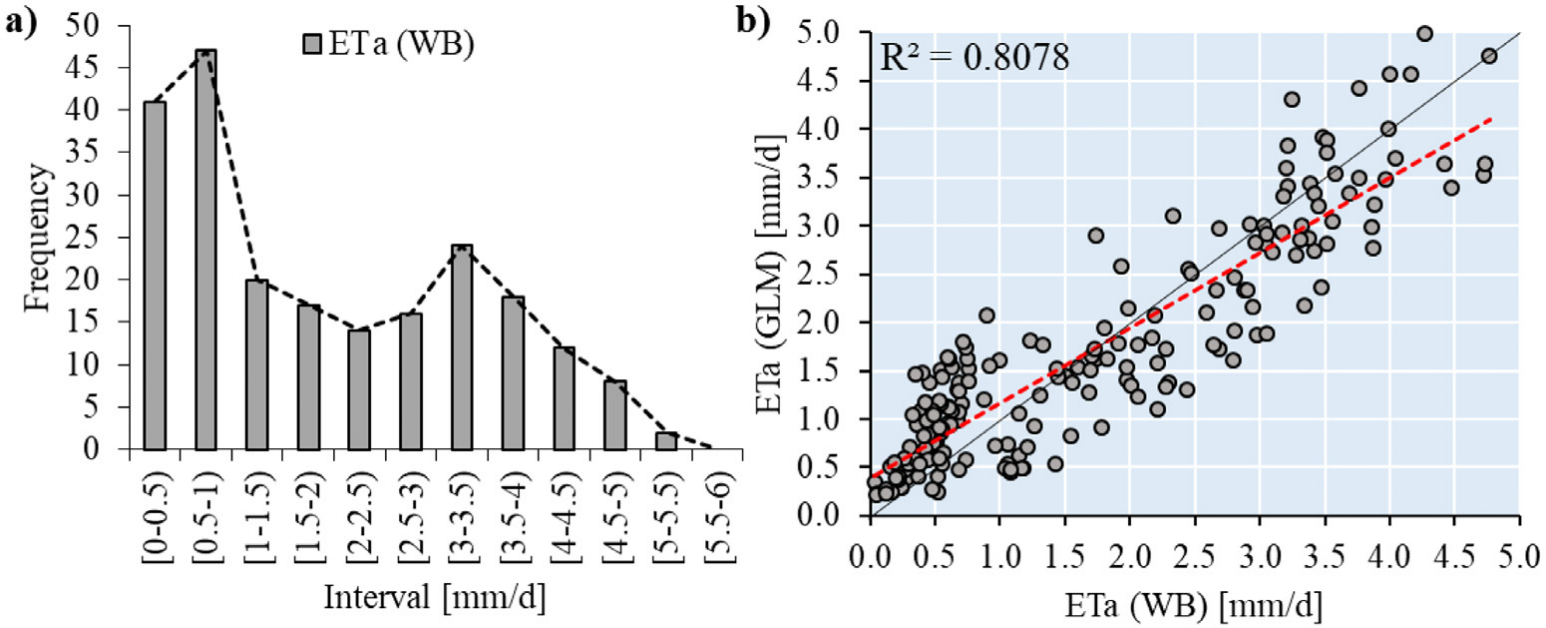
a) Frequency distribution of daily ETa obtained from water balance (WB), b) comparison between GLM and WB results at daily scale.

On the other hand, ETa measurements described a Poisson-Gamma distribution (Fig. 2a). This distribution has a complex probability density function. Thus, the distribution was adjusted to a Tweedie distribution [7]. The algorithm sklearn.linear_model.TweedieRegressor [6] allowed us to find the optimal link function to adjust the coefficients to the ETa distribution (Eq. 4). About the validation, the comparison between GLM results and the results obtained with the WB is included in Fig. 2b. About the performance of the model, the root mean square error (RMSE) was 0.577 mm/d, the Mean Absolute Error (MAE) was 0.473 mm/d, and the Determination Coefficient (R2) was 0.81.

The proposed GLM is suitable to follow a crop surface (from partially vegetated to full cover) with meteorological data measured near the canopy (2m height) and soil moisture measured in the root zone. It can be applied in other areas with data commonly obtained in typical energy balance or meteorological stations. The model showed the forcing variables of the soil-plant-atmosphere system that influence evapotranspiration.

Given the frequent limitation of ground data, the method can be used with satellite data, reanalysis or a combination of them. For example, the variables involved in Eq. 3 are available in the NASA-POWER dataset [8], which have a good correlation with field data [9,10]. Likewise, NDVI data are available in several missions (e.g. MODIS products MOD13C2, MOD13A1 version 6 [1,11]). It should be noted that a calibration model is needed to be applied in other areas with different biophysical characteristics to obtain good results, considering the variables involved in the method for those areas.

## Supplementary material *and/or* Additional information

All the tables mentioned in the text are included in https://data.mendeley.com/datasets/77r6w44xbp/draft?a=5d9be36a-ae9a-4c9f-b526-121d1d37b6eb

## Declaration of Competing Interest

The authors declare that they have no known competing financial interests or personal relationships that could have appeared to influence the work reported in this paper.
